# Socio-cultural beliefs and perceptions influencing diagnosis and treatment of breast cancer among women in Ghana: a systematic review

**DOI:** 10.1186/s12905-024-03106-y

**Published:** 2024-05-14

**Authors:** Agani Afaya, Emmanuel Anongeba Anaba, Victoria Bam, Richard Adongo Afaya, Ahmed-Rufai Yahaya, Abdul-Aziz Seidu, Bright Opoku Ahinkorah

**Affiliations:** 1 College of Nursing, Yonsei University, 50-1, Yonsei-ro, Seodaemun-gu, Seoul, 03722 South Korea; 2 Department of Nursing, School of Nursing and Midwifery, University of Health and Allied Sciences, Ho, Ghana; 3 Department of Population, Family and Reproductive Health, University of Ghana School of Public Health, Accra, Ghana; 4 Department of Nursing, Faculty of Allied Health Sciences, College of Health Sciences, Kwame Nkrumah University of Science and Technology, Kumasi, Ghana; 5 School of Nursing and Midwifery, Griffith University, Queensland, Australia; 6 Department of Internal Medicine, Tamale Teaching Hospital, Tamale, Ghana; 7 College of Public Health, Medical and Veterinary Sciences, James Cook University, Townsville, Australia; 8 School of Clinical Medicine, University of New South Wales, Sydney, Australia

**Keywords:** Breast cancer, Cultural, Religious and spiritual beliefs, Ghana, Systematic review

## Abstract

**Background:**

Breast cancer is currently the most commonly diagnosed cancer in Ghana and the leading cause of cancer mortality among women. Few published empirical evidence exist on cultural beliefs and perceptions about breast cancer diagnosis and treatment in Ghana. This systematic review sought to map evidence on the socio-cultural beliefs and perceptions influencing the diagnosis and treatment of breast cancer among Ghanaian women.

**Methods:**

This review was conducted following the methodological guideline of Joanna Briggs Institute and reported in accordance with the Preferred Reporting Items for Systematic reviews and Meta-Analyses. The literature search was conducted in PubMed, CINAHL via EBSCO*host*, PsycINFO, Web of Science, and Embase. Studies that were conducted on cultural, religious, and spiritual beliefs were included. The included studies were screened by title, abstract, and full text by three reviewers. Data were charted and results were presented in a narrative synthesis form.

**Results:**

After the title, abstract, and full-text screening, 15 studies were included. Three categories were identified after the synthesis of the charted data. The categories included: cultural, religious and spiritual beliefs and misconceptions about breast cancer. The cultural beliefs included ancestral punishment and curses from the gods for wrongdoing leading to breast cancer. Spiritual beliefs about breast cancer were attributed to spiritual or supernatural forces. People had the religious belief that breast cancer is a test from God and they resorted to prayers for healing. Some women perceived that breast cancer is caused by spider bites, heredity, extreme stress, trauma, infections, diet, or lifestyle.

**Conclusion:**

This study adduces evidence of the socio-cultural beliefs that impact on the diagnosis and treatment of breast cancer among women in Ghana. Taking into consideration the diverse cultural and traditional beliefs about breast cancer diagnosis and treatment, there is a compelling need to intensify nationwide public education on breast cancer to clarify the myths and misconceptions about the disease. We recommend the need to incorporate socio-cultural factors influencing breast cancer diagnosis and treatment into breast cancer awareness programs, education, and interventions in Ghana.

**Supplementary Information:**

The online version contains supplementary material available at 10.1186/s12905-024-03106-y.

## Introduction

Breast cancer is a global public health concern due to its increasing incidence coupled with the high mortality rate among women in low- and high-income countries [1]. In 2020, it was estimated that 2.3 million breast cancer cases were newly diagnosed with approximately 685,000 deaths globally [1]. In Ghana, breast cancer is the most commonly diagnosed cancer and the leading cause of cancer mortality among women [2]. In 2020, breast cancer accounted for approximately 31.8% of all cancer cases in Ghana [3].

Evidence shows that cultural factors such as conceptualizations of health, illness, beliefs, and values influence breast cancer screening among women in certain populations [4–6]. Breast cancer screening is reported to be relatively low among women living in Ghana. A nationwide study revealed that only 4.5% of Ghanaian women aged 50 years and older had undergone mammography screening [7]. The low levels of breast cancer screening lead to undetected breast cancer symptoms, contributing to the late-stage diagnosis of breast cancer and subsequent poorer outcomes and mortality [8]. There have been low levels of awareness and knowledge about breast cancer among women in Ghana [9]. Also, there is a lack of understanding of the perceptions and beliefs toward breast cancer diagnosis and treatment in Ghana.

Culture is considered a multidimensional set of shared beliefs and socially transmitted ideologies about the world, which are passed on from generation to generation [10, 11]. Cultural beliefs within certain communities across the globe are considered a determinant of health risk perceptions and behaviors in promoting or seeking health care in diverse populations [12]. In traditional Ghanaian communities, good health is recognized as a suitable relationship between the living and the dead and being in harmony with the individuals’ environment. Thus, disease is conceptualized as a malfunctioning of the body system which is probably due to a lack of harmony with supernatural/ancestral forces [13]. This belief influences how diseases are treated and the steps taken to manage the disease and ultimately how the disease is experienced [13, 14]. Cultural beliefs connected to breast cancer are among the key determinants in women’s decision-making regarding breast cancer screening practices in traditional societies [14, 15]. In most Ghanaian communities, breast cancer is believed to be associated with supernatural powers, hence, women seek alternative treatments (healing/prayer camps) first and only report to health facilities in advanced stages of breast cancer [16].

It is therefore important to consider how socio-cultural factors impact breast cancer diagnosis and treatment because these factors influence cancer care in resource-limited settings. To the best of our knowledge, no review has been conducted in Ghana specifically to address the cultural, religious, and spiritual beliefs influencing timely diagnosis and treatment of breast cancer among women. To fill this gap, this systematic review sought to map evidence on the cultural beliefs and perceptions that influence the timely diagnosis and treatment of breast cancer among women.

## Methods

This systematic review was conducted following the updated methodological guideline of Joanna Briggs Institute (JBI) [17, 18] and reported in accordance with the Preferred Reporting Items for Systematic reviews and Meta-Analyses (PRISMA) statement. The updated JBI methodological guidance regarding conducting a mixed methods systematic review recommends that reviewers use a convergent approach to synthesize and integrate both qualitative and quantitative studies [18]. Therefore, using a mixed methods systematic review involving both quantitative and qualitative studies was deemed the most appropriate study design because this is the first evidence synthesis on the cultural, religious, and spiritual beliefs that influence breast cancer diagnosis and treatment in Ghana.

### Inclusion and exclusion criteria

- Studies conducted among women and explored the cultural beliefs and perceptions about breast cancer were included.
- Studies that were only limited to Ghanaian communities were included.
- Empirical studies published in peer-review journals.
- Observational studies, using qualitative and/or quantitative methods were also included.
- The exclusion criteria involved review studies, conference papers, editorials and abstracts.
- Studies published before 2012 were also excluded.

### Search strategy

This review adopted the triple-step search strategy proposed by the JBI for all types of reviews [19]. The first step involved an initial limited search in PubMed for already existing published research articles on sociocultural beliefs and perceptions about breast cancer in Ghana. The initial limited search ensured the identification of relevant keywords used in developing the preliminary search terms. Step two involved a formal search after finalizing and combining the following keywords (‘breast cancer’, ‘cultural beliefs’, ‘religious beliefs’, ‘traditional beliefs’, ‘perception’, and ‘Ghana’) using Boolean operators. A comprehensive search was conducted in PubMed, CINAHL via EBSCO*host*, PsycINFO, Web of Science, and Embase from 2012–2022. The final step involved manual tracing of the reference list of studies for additional studies. This was done up to the point of saturation where no new information emanated from the subsequent manual search of articles.

### Study selection

Following the searches, the identified records were exported into EndNote 2020 reference manager for duplicate removal. After the duplicate removal, the reviewers ensured consistency in screening through the following process: (1) joint screening by two reviewers was conducted until they felt confident to start independent screening, (2) independent blinded screening of titles/abstracts followed by a meeting and discussion of discrepancies and (3) repetition of step 2 until an acceptable agreement was met. Following the screening of the titles/abstracts, full-text review was conducted following a two-step process. The first step involved two reviewers who screened all the articles identified after the title/abstract screening. Thereafter, two independent reviewers assessed the full-text articles for inclusion or exclusion. In the course of the full-text screening, any disagreements that emerged were discussed for consensus. Throughout the screening of the abstracts, full-texts, and data extraction, the reviewers regularly met to discuss and solve emerging issues.

### Data extraction

A data extraction form was developed in line with the aim of this review. Two authors independently extracted the relevant information from the included articles. The following information was extracted from the articles: first author’s name, year of publication, study location, study type, aim, study population, and key findings. Disagreements during the data extraction process were resolved by a discussion and where a resolution was not reachable, the last author resolved it through further adjudication. Study selection and data extraction were conducted manually.

### Data analysis

A convergent integrated approach [20] was employed to transform the data into narrative form because the extracted information was from quantitative and qualitative studies. The analysis followed JBI recommendation where we qualitized quantitative data for data transformation because this is less prone to error when codified than when qualitative data is given numerical values. Qualitizing entails taking data from quantitative studies, translating or converting it into textual descriptions so that it can be integrated with qualitative data, and providing a narrative interpretation of the quantitative results [18]. Following the convergent synthesis of the transformed data, the reviewers undertook repeated, detailed examination of the assembled data to identify categories on the basis of similarity in meaning [18]. Out of these, three categories were derived from the analysis.

### Assessment of methodological quality

Using the Mixed Methods Appraisal Tool (MMAT)  version 2018, two researchers (AA and RAA) evaluated each included study’s quality separately [21]. After discussing disagreements between the two reviewers (AA and RAA), BOA helped to forge a consensus. Methodological quality standards for evaluating research using mixed methodologies, quantitative, and qualitative approaches are included in the MMAT. The MMAT assesses the suitability of the research objective, study design, technique, participant recruitment, data collection, data analysis, results presentation, author comments, and conclusions. Hong et al. [21] discourages the overall quality scoring of the included studies, therefore, the methodological quality of the studies was evaluated using the recommended guidelines.

**Fig. 1:**
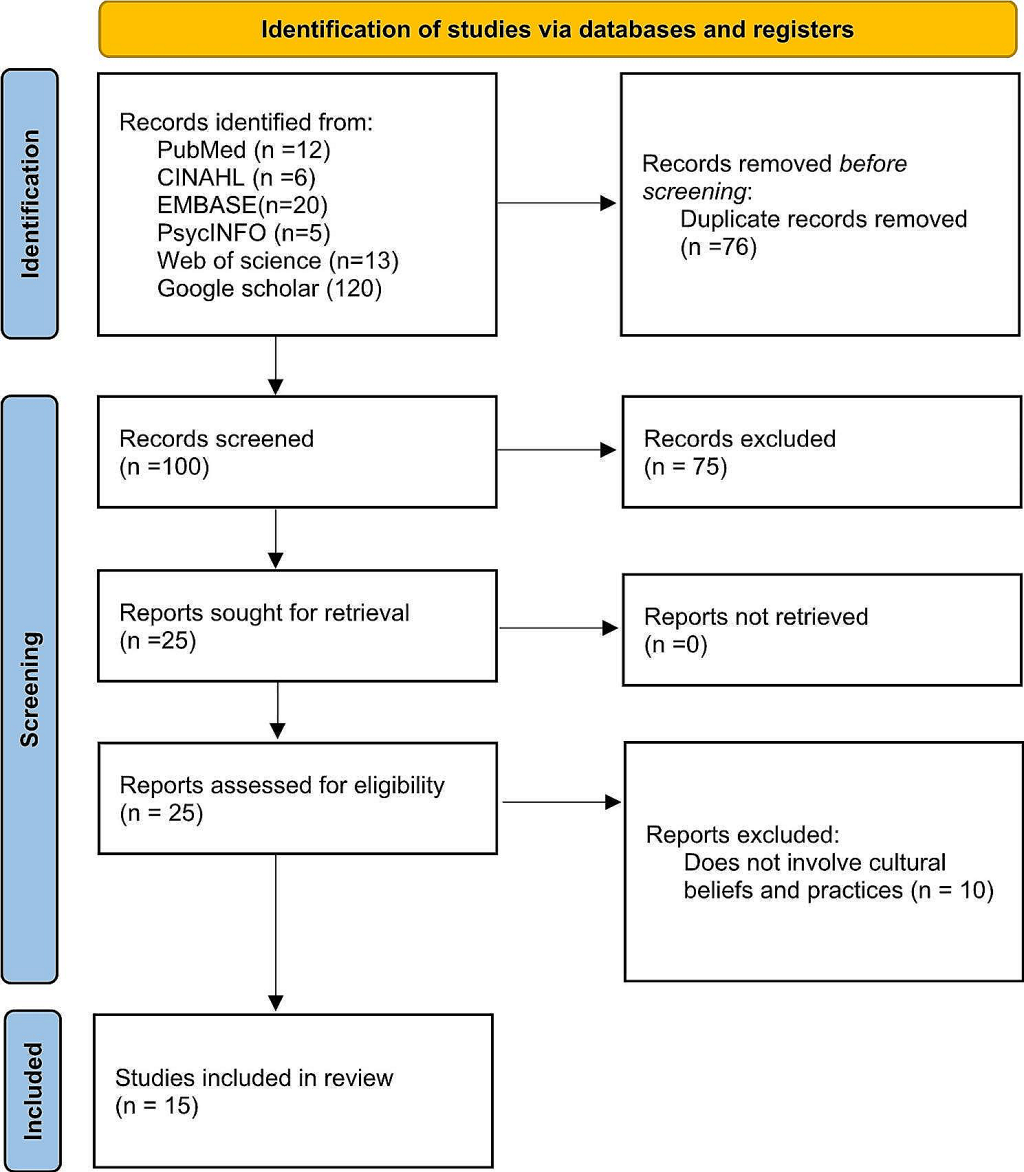
Flow Chart of evidence selection

## Results

### Literature search

Our search yielded a total of 176 records from the electronic databases. After duplicates were automatically removed through the EndNote (*n* = 76), 100 records were reviewed independently by two authors based on the title and abstract. Records that did not meet the inclusion (*n* = 75) were removed after holding discussions to identify discrepancies in the review process. Thereafter, full texts of the remaining 25 articles were assessed for eligibility. Hand-search of the included study references yielded no results. In total, we included 15 studies [22–36]. The article selection process is shown in the PRISMA flow diagram (Fig. 1).

### Characteristics of the included studies and quality

The majority of the studies [22–24, 26–35] were conducted in the southern part of Ghana where there are better health infrastructures compared to the northern part of Ghana. Eight of the included studies were qualitative while the rest employed quantitative study designs. The summary of the characteristics of the 15 studies is shown in Table 1. The appraisal of the included studies was assessed using the MMAT. All the studies were included, and none were excluded due to poor methodological quality. All 15 studies met the screening criteria and provided clear research questions. The studies included clearly stated and described research design, and target population, and used appropriate measurements.

**Table 1:** Characteristics of included studies

First Author (year)	Study location	Study aim	Study type	Population/Sample	Key findings	
					Cultural/religious beliefs & perception	
Addae-Korankye (2016) [22]	Tamale metropolis, Northern region	To assess the extent of awareness or knowledge of breast cancer among women in Tamale metropolis in Ghana.	Quantitative study	120 women	Participants believe the causes of breast cancer (BC) are curses or punishment by gods for sins committed, hereditary, dietary or lifestyle, stress/trauma, and infections.	
Agbokey (2014) [23]	Kumasi, Ashanti region	1. To explore breast cancer patient’s beliefs about the causes of breast cancer. 2. To document the various sources of care that breast cancer patients resort to in seeking health care for breast cancer. 3. To document reasons for delayed medical care for breast cancer. 4. To explore the sources of information about treatment options for breast cancer.	Qualitative study	The total respondents were 35: Breast Cancer patients (20), Health workers (5), Caregivers (8), Herbalists (2)	Respondent perceived breast cancer to be an extremely dangerous, terrible, and fatal disease which spreads extremely fast and kills instantly. A punishment from the ancestors and the gods for refusal to give birth and continue the human race. *spiritual* babies to suckle their breasts to give breast cancer. Breast cancer is caused by witches, insect (spider) bites, and men’s frequent sucking of women‘s breasts. Heredity and lifestyles such as eating fatty foods as predisposing to breast cancer. Herbs can cure the disease. The monies kept by the breast as well as keeping phones on the breast are all pathways to or causes of getting breast cancer.	
Agbokey (2019) [24]	Komfo Anokye Teaching Hospital, Ashanti region	To explore the health seeking behavior of breast cancer patients and their knowledge of breast cancer in a breast cancer management.	Qualitative study	35 respondents: Breast Cancer patients (20), Health workers (5), Caregivers (8), Herbalists (2)	Perceived that breast cancer is a punishment meted out by their ancestors or their gods for not having children to save the human race from extinction. Attributed breast cancer condition to a spider bite that led to itching around the nipple and nipple discharge. Likened the growth of breast cancer to that of uterine fibroid.	
Asobayire (2015) [25]	Kassena-Nankana district, Upper East region	To ascertain how societal perceptions and attitudes influence women’s awareness of breast cancer.	Qualitative study	10 participants: 6 farmers, 2 traders, and 2 teachers.	Participants commonly thought that when a woman is unable to breastfeed after giving birth, this can lead to a swollen breast or ‘ngwoom pongwa’ (a boil in the breast). Large breast sizes have the propensity to be at risk of developing long-term breast cancer. Participants perceived that breast cancer is a mere lump or boil. Breast cancer is often viewed as a kind of punishment.	
Asoogo (2015) [26]	A tertiary hospital in Kumasi, Ashanti region	To describe the factors which contribute to the late presentation of Ghanaian women with breast cancer for health care at a tertiary hospital in Kumasi, Ghana.	Qualitative study	30 breast cancer patients	We contacted a traditional healer and prayed to the ancestors for traditional medicines to work. Women went to prayer camp for almost the whole year for spiritual healing. Participants were taken to traditional doctors.	
Azumah (2017) [27]	Asokore in the Sekyere East District in the Ashanti Region	To examine the community knowledge, perception, and attitude toward breast cancer in Asokore in the Sekyere East District.	Quantitative study	97 women.	The majority of the respondents perceived that they do not have any risk factors for breast cancer 25.8% (25) of the respondents felt that breast cancer is a curable disease, 54.6% (53) of the respondents felt that breast cancer is not a curable disease and 19.6% of the respondents could not tell whether breast cancer was a curable disease or not. The majority of the respondents felt that breast cancer was not a curable disease. The respondents have the perception that once a women contract breast cancer, it cannot be cured. The majority of the respondents indicated that they will not see a male doctor examine their breasts in case of breast cancer. Women in the study area do not believe that breast cancer occurs or more commonly occurs among women of old age.	
Boafo (2020) [28]	University of Ghana, located in Accra, the capital of Ghana	To explored the knowledge levels and health beliefs of university students on breast cancer and BSE, explored the prevalence of BSE among university students, and examined the predictors of BSE among university students in Ghana.	Quantitative study	308 female nonmedical students	43.4% of the university students sampled erroneously believed that breast cancer can be caused by a man sucking a woman’s breasts. Participants had moderate knowledge of breast cancer. A substantial proportion of them (68%) believed that younger women of their ages do not get cancer.	
Bonsu (2019) [29]	Komfo Anokye Teaching Hospital, Kumasi Ahanti region	To explore the reasons for delayed presentation in Ghanaian women with breast cancer.	Qualitative study	11 advanced breast cancer patients	God’s punishment and spiritual attacks because of specific actions such as extramarital immoral life. Believed the cause of the disease was an attack by a family member through supernatural power intended to destroy or kill her.	
Dadzi (2019) [30]	Akatsi South District of the Volta region	To examine awareness and knowledge of breast self-examination as well as the practice of breast self-examination among rural women.	Quantitative study	385 women	177(46.1%) of the participants believed breast cancer is the most common cancer in women. Participants mentioned that family history and women who did not breastfeed, contraceptive use, being a woman, and obesity or overweight are risk factors for breast cancer. Participants believed breast cancer is curable if detected early. Other participants believed that breast cancer is not curable but can be controlled.	
Iddrisu (2021) [31]	University of Ghana hospital, 37 Military hospital, and Ridge Hospital. Greater Accra	To explore the socioeconomic impact of breast cancer on young women in Ghana.	Qualitative study	12 breast cancer patients	A dangerous disease, that kills faster. Cancer deadly, Evil disease, and a test from God. Contagious disease and very transmissible	
Kugbey (2020) [32]	Radiotherapy and Nuclear Medicine Department of the Korle-Bu Teaching Hospital (KBTH) in Ghana, West Africa	To explore illness perception and coping strategies used by women living with breast cancer in Ghana.	Qualitative study	11 women	A substantial number of the women stated that they did not know what causes breast cancer, others mentioned supernatural forces, physiology, and stress as the probable causes of their illness. Most of participants believed that their disease can be completely cured, and this belief was rooted in their faith in God and the medical treatments.	
Opoku et al., (2012) [33]	Two Ghanaian cities, Accra and Sunyani	To determine population-based rates of reported breast cancer screening and assess breast cancer-related knowledge, attitudes, beliefs among Ghanaian women and explore their relation to screening practices in the study areas.	Quantitative study	500 women	A prominent misconception held by 20% of the respondents was to the effect that coins put in the brassieres can increase a woman’s risk for the disease. Respondents’ attitudes towards the disease include fear which was linked to death in most cases; denial and guilt; as well as the spiritual and supernatural attributes of the disease.	
Osei et al., (2021) [34]	University of Health and Allied Sciences (UHAS) of Ghana, Volta region	To explore the perceived risk of breast cancer among female undergraduate students in Ghana and to examine factors associated with perceived risk.	Quantitative study	385 female undergraduate students	Breast perceived risks found in the study are advancing age, knowledge of someone with breast cancer, family history of breast cancer, history of breast cancer screening, intention to perform breast self-examination	
Osei-afriyie (2021) [35]	University of Health and Allied Sciences (UHAS), Volta Region, Ghana	To explore breast cancer awareness, selected risk factors, and screening practices among female undergraduate students, to provide information for the control, prevention, and preliminary treatment of the disease.	Quantitative study	385 students	Family history of breast cancer, genetics, female sex, and individual lifestyle were the most frequently perceived risk factors for breast cancer. Putting money in the brassiere was perceived as a potential risk factor for breast cancer by more than a third of the respondence.	
Salisu (2022) [36]	Tamale Teaching Hospital, Northern region	To explore how breast cancer patients’ personal beliefs and ideas influence their decision to refuse medical treatment.	Qualitative study	13 breast cancer patients	Women taken to traditional healers for breast cancer treatment and the women had so much faith in the traditional healer. Women believed that their cancers were spiritual attacks from neighbors and needed to be cast out. Some women believed that it was their destiny to have breast cancer	

### Cultural beliefs

Breast cancer is believed by some sections of Ghanaians to be a curse or a punishment from the lesser gods for sins committed by the individual [22]. Some women believed that an extra-marital immoral lifestyle provokes God’s retribution for breast cancer development [29]. Some people believed that it is an ancestral punishment for the woman’s refusal to give birth in order to continue the ancestral lineage [23] and because of this, they are given spiritual babies to suckle the breast which then causes cancer [23]. It is also believed some women have been pronounced cursed due to some wrongdoings [25]. Due to the cultural belief, some women prayed to their ancestors so that traditional medicine will heal them of the breast cancer [26].

*“…when it started, my uncles came to my aid, they took me to the village to see a “Tim Lana” (referring to a traditional healer). He was very good. He told me everything about my problem. So, there was no need for visiting the hospital…”* [36].

### Spiritual and religious beliefs

Some studies in Greater Accra, Tamale, and Kumasi indicated that breast cancer was a spiritual attack from humans or family members that sought to kill them while some believe it emanated from evil forces [29, 31, 36]. Participants in some studies indicated that breast cancer is attributed to some spiritual or supernatural forces [32, 33, 36] and can only be cured through spiritual means [33]. Due to the spiritual beliefs, some women went to traditional healers for treatment [26, 36]. A study in the northern part of Ghana revealed that women who suffer from breast cancer are witches and have used their breasts for ritual purposes [25] while in the southern part of Ghana some participants believed that breast cancer is caused by witches [22]. For example, a narration from a participant stated:

*“I believe my condition is spiritual and I realized it is coming from my mother’s side”* [31].

*“The problem is that my disease is a spiritual attack, so it has to be treated spiritually; the hospital drugs cannot get this out of me…”* [36].

Some studies in the southern and northern part of Ghana stated that participants had a religious belief that the disease was a test from God and resulted in prayers for healing [31, 36] and also believed that God had the supernatural powers to miraculously melt the breast lump [29, 32] and completely cure them [32]. Some women also believed that it was their fate to get breast cancer [36]. Due to these religious beliefs some women had to resort to prayer camps for healing which leads to delay in diagnosis and treatment of breast cancer [26].

### Misconceptions about breast cancer

Some women perceived that breast cancer is caused by spider bites [24], heredity, extreme stress [22, 32], trauma, infections [22], diet, or lifestyle [22, 35]. Some perceived risk factors of breast cancer as stated by some women included non-breastfeeding women, obesity, or overweight [25, 30, 33], and contraceptive use [30]. Some women had the perception that male health practitioners would not be allowed to examine or see their breasts while some preferred male doctors to examine their breasts [27]. A study in Accra conducted among female nonmedical students revealed that suckling the breast by a male caused breast cancer [28]. It is also perceived that putting money in the brassieres could be a possible cause of breast cancer among females [23, 35]. A study by Iddrisu et al. [31] and Agbokey [23] revealed that breast cancer is a disgraceful disease, dangerous, and a fast killer. Some people also believed that breast cancer can be cured [27, 32] by herbal treatment or medicine [25] while some believed that it is not curable [27]. Some people also believed that breast cancer was contagious and transmissible and avoided sharing equipment with breast cancer survivors [31]. A breast cancer survivor narrated:

*“…my mum believes the disease can be transmitted so she does not allow me to eat with my son. I have separate bowls, spoons, and cups from that of the family…”* [31].

## Discussion

This study reviews the existing literature on socio-cultural beliefs influencing the timely diagnosis and treatment of breast cancer among women, and this revealed diverse cultural, spiritual, and religious beliefs across the regions of Ghana. The current findings emphasize critical issues that lead to misguidance and share ignorance about breast cancer and its treatment among a section of Ghanaian communities which is rooted in their personal beliefs. Cultural beliefs are key in the decision-making process for the treatment of ailments depending on their knowledge level about the condition. This could probably lead to making the right decision or the wrong treatment decision. The diverse cultural, spiritual, and religious beliefs about breast cancer could affect the health seeking behavior of women diagnosed with breast cancer within the Ghanaian communities.

Consistent with a systematic review findings [13] it is believed that breast cancer emanates as a result of supernatural forces, curses, and punishment from lesser gods/ancestors for wrongdoings. Though not all Africans hold this traditional belief in ancestral spirits, some believe that health and illness are in the hands of a higher power such as God or Allah [13]. Hence, in most African communities it is common practice to seek traditional medicine for the treatment of diseases which is in line with their beliefs [37]. Due to the cultural/traditional belief systems and practices, most women report to health facilities with advanced stages of breast cancer which adversely impacts the breast cancer diagnosis and treatment [36]. Most women resort to traditional or spiritual healing because this method of treatment combines body, soul, and spirit. In some African settings, traditional healers are trusted to treat diseases including cancer because women believe they look for both scientific and metaphysical causes of the disease. It is possible that breast cancer patients who combine both traditional and modern methods of treatment may experience treatment interference. This dual approach can impact treatment effectiveness and lead to adverse effects or complications. The provision of culturally sensitive care by recognizing unique cultural, religious, and social beliefs and practices is of paramount importance for early detection and treatment of breast cancer among women [38–40]. Globally, women’s cultural beliefs and perceptions towards breast cancer should be examined to optimize timely breast cancer diagnosis and treatment.

Religious fanaticism coupled with lack of knowledge about the disease condition could impede the utilization of medical treatment, especially when religious beliefs impact negatively on people’s health-seeking behaviors [36]. A study in Nigeria revealed that religious beliefs about breast cancer were observed to be a barrier to breast cancer screening among women [41]. This review found that some women in the southern part of Ghana believed that breast cancer was a test from God and resorted to prayers because they believed that God had supernatural powers to heal them from the disease. Though religious beliefs are considered to be a source of spiritual strength and help people to cope with the disease, the religious misconceptions, and mistaken beliefs are thought to contribute to delayed heath-seeking attitudes and lack of breast cancer screening among women [42]. In the current review, it was reported that some women stayed in prayer camps for almost one year seeking healing and later reported to health facilities with advanced breast cancer which has dire consequences on the survival rate of women. Efforts to sensitize women and religious leaders about the early presentation of breast disease to health facilities for diagnosis and treatment would be key to reduce the number of breast cancer cases detained in religious camps. It is also imperative for religious bodies to discuss health related issues including breast cancer to create much awareness about the condition.

This review identified varied perceptions of breast cancer where breast cancer has been attributed to spider bites and putting money in the brassieres among others. Some believed that breast cancer was a contagious and transmissible disease. These findings show poor knowledge level among women concerning breast cancer. Even though in this review most women had heard or were aware of breast cancer, the varied perceptions about breast cancer suggests low knowledge level of breast cancer. The low knowledge level of breast cancer among women have been associated with late presentation of breast cancer to health facilities [40]. Women presenting to health facilities with advanced stage breast cancer have been associated with low survival rate in the African region as compared to high income countries [43]. A study conducted in Ghana revealed that the breast cancer survival rate among women was below 50% which was probably due to late presentation and lack of breast cancer screening [44]. We recommend intensification of public health education campaigns on breast cancer in order to improve women’s knowledge of the disease which will subsequently enhance early presentation, diagnosis, and treatment.

### Implication for policy and practice

Metaphors such as spider bites, supernatural forces, witchcraft, and many other beliefs are associated with breast cancer in Ghana which impact the understanding of the disease and whether or not to seek medical treatment. Therefore, culturally sensitive intervention programs targeted at improving breast cancer awareness among women, religious and traditional leaders are imperative. These intervention programs could entail community engagement, workshops, or educational materials tailored to address specific cultural beliefs and misconceptions.

Taking into consideration the diverse cultural beliefs about breast cancer, there is a compelling need for nationwide public education on breast cancer to clarify the myths and misconceptions about the disease. The education program should be culturally tailored to address the myths and misconceptions. It is important that considerations are given to these issues, not only focusing on how these issues affect women’s lives post-treatment but also on how these issues can be resolved to improve diagnosis and treatment of the disease. We recommend that socio-cultural factors influencing breast cancer diagnosis and treatment should be incorporated into breast cancer awareness programs, education, and intervention programs in Ghana. We believe these would help inform women and encourage them to report to health facilities early with breast cancer symptoms to initiate timely diagnosis and treatment to improve the outcomes of the disease in Ghana.

Further research is required to explore appropriate and effective multidimensional culturally sensitive intervention research that integrates cultural beliefs and breast cancer treatment especially, in different Ghanaian communities.

### Strengths and limitations of the study

This study has several strengths, one major strength is the extensive and comprehensive search in various electronic databases following the methodological guideline of JBI and reported in accordance with the PRISMA guidelines. Also, the inclusion of both qualitative and quantitative studies, allowed for a more comprehensive understanding of the socio-cultural beliefs influencing breast cancer diagnosis and treatment in Ghana.

The review considered only published studies and possibly may have overlooked unpublished or gray literature that could contribute to a more comprehensive understanding of the subject matter. Most of the studies were concentrated in the southern part of Ghana and therefore the results might not represent all the regions in Ghana.

## Conclusion

This study adduces evidence on the socio-cultural beliefs that impact diagnosis and treatment of breast cancer among women in Ghana. As policy makers, clinicians and other stakeholders strive to improve breast cancer diagnosis and treatment, there is a need to address the socio-cultural beliefs to improve breast cancer outcomes in Ghana and potentially reduce breast cancer-related mortality.

### Electronic supplementary material

Below is the link to the electronic supplementary material.

Supplementary Material 1

## Data Availability

The datasets used and/or analysed during the current study are available from the corresponding author on reasonable request.
