# Supplementary material for: Socio-cultural beliefs and perceptions influencing diagnosis and treatment of breast cancer among women in Ghana: a systematic review
Source: BMC Womens Health. 2024 May 14;24:288. doi: 10.1186/s12905-024-03106-y (PMC11092234; doi:10.1186/s12905-024-03106-y)
Supplement: Supplementary file 1 — Supplementary Material 1 [file 12905_2024_3106_MOESM1_ESM.docx]

**Appendix**  **sample of search strategies**

| **Databases** | **No** | **Search terms** | **Results** |
| --- | --- | --- | --- |
| **PubMed** | 1 | ("Breast Neoplasms"[MeSH Terms] OR "Breast Neoplasms"[All Fields] OR "Breast cancer"[All Fields] OR "Breast Neoplasia"[All Fields] OR "Breast Tumor"[All Fields]) AND (2012:2022[pdat]) | 215,576 |
|  | 1 | "Breast Neoplasms"[MeSH Terms] OR "Breast Neoplasms" OR "Breast cancer" OR "Breast Neoplasia" OR "Breast Tumor" |  |
|  | 1 | ("cultural belief*"[All Fields] OR "traditional belief*"[All Fields] OR "religious belief*"[All Fields] OR "spiritual belief*"[All Fields] OR "percept"[All Fields] OR "perceptibility"[All Fields] OR "perceptible"[All Fields] OR "perception"[MeSH Terms] OR "perception"[All Fields] OR "perceptions"[All Fields] OR "perceptional"[All Fields] OR "perceptive"[All Fields] OR "perceptiveness"[All Fields] OR "percepts"[All Fields]) AND (2012:2022[pdat]) | 341,134 |
|  | 2 | “Cultural belief*” OR “traditional belief*” OR “religious belief*” OR “spiritual belief*” OR perception | 341,134 |
|  | 3 | ("ghana"[MeSH Terms] OR "ghana"[All Fields] OR "ghana s"[All Fields] OR "ghanaian"[All Fields] OR "ghanaians"[All Fields]) AND (2012:2022[pdat]) | 17,411 |
|  | 3 | "ghana"[MeSH Terms] OR "ghana" OR "ghana s" OR "ghanaian" OR "ghanaians" |  |
|  | 4 | **#1 AND #2 AND #3** | 12 |
|  | 5 |  |  |
| **Web of Science** | 1 | TS=(‘Breast cancer’ OR ‘Breast Neoplasia’ OR ‘Breast Neoplasms’ OR ‘Breast Neoplasm’ OR ‘Breast Tumor’) | **375,930** |
|  | 2 | TS=(“Cultural belief*” OR “traditional belief*” OR “religious belief*” OR “spiritual belief*” OR perception*) | **522,739** |
|  | 3 | TS=(Ghana OR Ghanaian) | **23,280** |
|  |  |  |  |
|  | 4 | **#1 AND #2 AND #3** | **13** |
| **CINAHL** | 1 | ("Breast cancer" OR "Breast Neoplasia" OR "Breast Neoplasms” OR "Breast Neoplasm" OR “Breast Tumor”) | 1,239 |
|  | 2 | (“Cultural belief*” OR “traditional belief*” OR “religious belief*” OR “spiritual belief*” OR perception*) | 121,509 |
|  | 3 | (Ghana OR Ghanaian) | 4,964 |
|  |  |  |  |
|  | 4 | **#1 AND #2 AND #3** | 6 |
| **EMBASE** | 1 | (**'breast cancer'**/exp OR **'breast cancer'** OR **'breast neoplasia'**/exp OR **'breast neoplasia'** OR **'breast neoplasms'**/exp OR **'breast neoplasms'** OR **'breast neoplasm'**/exp OR **'breast neoplasm'** OR **'breast tumor'**/exp OR **'breast tumor'**) AND [2012-2022]/py | 403,865 |
|  | 2 | (**'cultural belief*'** OR **'traditional belief*'** OR **'religious belief*'** OR **'spiritual belief*'** OR **perception***) AND [2012-2022]/py | 310,236 |
|  | 3 | (**'ghana'**/exp OR **'ghana'** OR **'ghanaian'**/exp OR **'ghanaian'**) AND [2012-2022]/py | 20981 |
|  |  |  |  |
|  | 4 | **#1 AND #2 AND #3** | 20 |
| **PsycINFO** | 1 | ("Breast cancer" OR "Breast Neoplasia" OR "Breast Neoplasms" OR "Breast Neoplasm" OR "Breast Tumor") | 7,771 |
|  | 2 | (“Cultural belief*” OR “traditional belief*” OR “religious belief*” OR “spiritual belief*” OR perception*) | 238,315 |
|  | 3 | (ghana OR Ghanaian) | 3,586 |
|  |  | #1AND #2 AND #3 | 5 |
|  |  |  |  |
|  |  |  |  |
